# Species diversity of non-tuberculous mycobacteria isolated from humans, livestock and wildlife in the Serengeti ecosystem, Tanzania

**DOI:** 10.1186/s12879-014-0616-y

**Published:** 2014-11-18

**Authors:** Bugwesa Z Katale, Erasto V Mbugi, Louise Botha, Julius D Keyyu, Sharon Kendall, Hazel M Dockrell, Anita L Michel, Rudovick R Kazwala, Mark M Rweyemamu, Paul van Helden, Mecky I Matee

**Affiliations:** Department of Microbiology and Immunology, School of Medicine, Muhimbili University of Health and Allied Sciences (MUHAS), Dar Es Salaam, Tanzania; Tanzania Wildlife Research Institute (TAWIRI), Arusha, Tanzania; Centre for Emerging, Endemic and Exotic diseases, Royal Veterinary College (RVC), Hawkshead Lane, North Mymms, Hatfield, Hertfordshire AL9 7TA UK; Department of Immunology and infection, London School of Hygiene and Tropical Medicine (LSHTM), London, UK; Department of Veterinary Tropical Diseases, Faculty of Veterinary Science, University of Pretoria, Onderstepoort, Pretoria, South Africa; Southern African Centre for Infectious Diseases Surveillance (SACIDS), Sokoine University of Agriculture (SUA), Chuo Kikuu, Morogoro, Tanzania; Department of Veterinary Medicine and Public Health, Sokoine University of Agriculture (SUA), Faculty of Veterinary Medicine, Chuo Kikuu, Morogoro, Tanzania; DST/NRF Centre of Excellence for Biomedical Tuberculosis Research/MRC Centre for TB Research, Division of Molecular Biology and Human Genetics, Faculty of Medicine and Health Sciences, Stellenbosch University, Tygerberg, Cape Town, South Africa

**Keywords:** Non-tuberculous mycobacteria, Species diversity, Human-animal interface, Serengeti ecosystem

## Abstract

**Background:**

Non-tuberculous mycobacteria (NTM), which are ubiquitous micro-organisms occurring in humans, animals and the environment, sometimes receive public health and veterinary attention as opportunistic disease-causing agents. In Tanzania, there is limited information regarding the diversity of NTM species, particularly at the human-livestock-wildlife interface such as the Serengeti ecosystem, where potential for cross species infection or transmission may exist.

**Methods:**

Mycobacterial DNA was extracted from cultured isolates obtained from sputum samples of 472 suspect TB patients and 606 tissues from wildlife species and indigenous cattle. Multiplex PCR was used to differentiate NTM from *Mycobacterium tuberculosis* complex (MTBC) members. NTM were further identified to species level by nucleotide sequencing of the 16S rRNA gene.

**Results:**

A total of fifty five (55) NTM isolates representing 16 mycobacterial species and 5 isolates belonging to the MTBC were detected. Overall, *Mycobacterium intracellulare* which was isolated from human, cattle and wildlife, was the most frequently isolated species (20 isolates, 36.4%) followed by *M. lentiflavum* (11 isolates, 20%), *M. fortuitum* (4 isolates, 7.3%) and *M. chelonae-abscessus* group (3 isolates, 5.5%). In terms of hosts, 36 isolates were from cattle and 12 from humans, the balance being found in various wildlife species.

**Conclusion:**

This study reveals a diversity of NTM species in the Serengeti ecosystem, some of which have potential for causing disease in animals and humans. The isolation of NTM from tuberculosis-like lesions in the absence of MTBC calls for further research to elucidate their actual role in causing disease. We are also suggesting a one health approach in identifying risk factors for and possible transmission mechanisms of the NTM in the agro-pastoral communities in the Serengeti ecosystem.

**Electronic supplementary material:**

The online version of this article (doi:10.1186/s12879-014-0616-y) contains supplementary material, which is available to authorized users.

## Background

Non-tuberculous mycobacteria (NTM) are saprophytic acid-fast bacilli, which are present in water, soil and biofilms [1]-[3]. Various names such as anonymous, atypical, environmental, opportunistic and mycobacteria other than tubercle bacilli (MOTT) have been used interchangeably referring to NTM [4]. In contrast to obligate parasites such as *M. tuberculosis* and *M. leprae,* NTM are present in the environment as saprophytes causing disease opportunistically [2],[3]. Similarities of NTM isolates have been found in humans and animals, soil and natural open water sources, implying a possible transmission via the environment [3].

**Figure 1:**
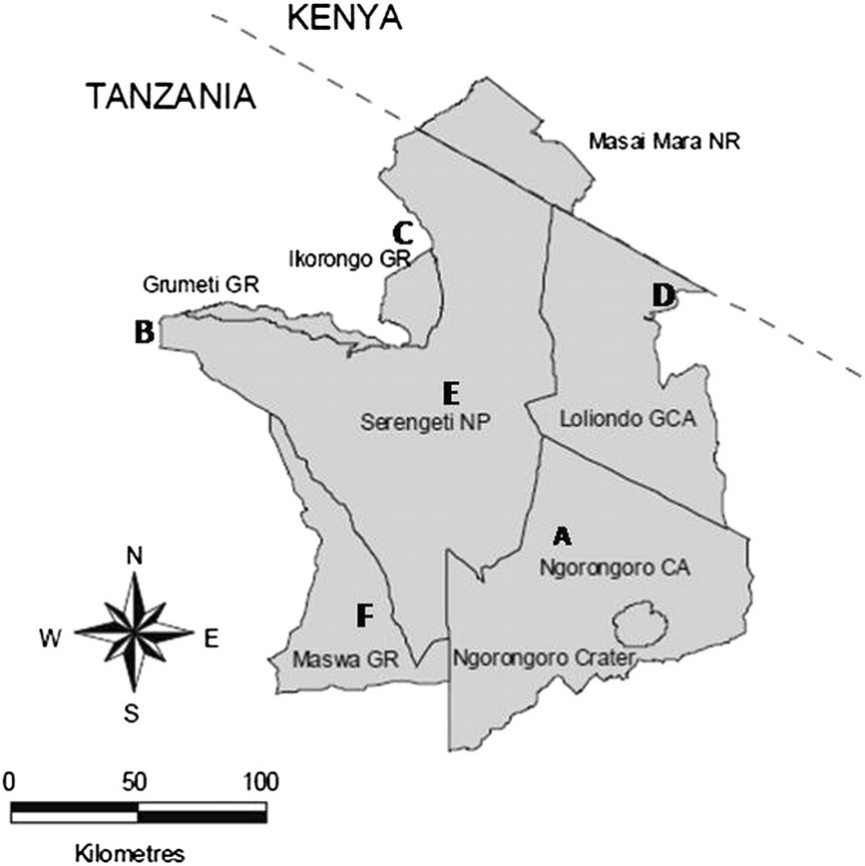
**Map of the Serengeti ecosystem which shows study sites where tissues from animals and sputum samples from TB suspect patients were collected; A; Endulen Health centre, B; Bunda [Bunda district headquarter (HQ)], C; Mugumu (Serengeti district HQ), D; Waso-Loliondo (Ngorongoro district HQ), E; Serengeti National Park, F; Maswa Game Reserve.**

NTM play a significant role as sources of human and animal infections [2],[5] with varied clinical manifestations [3] and should be a cause of concern in countries with high prevalence of human immunodeficiency virus (HIV) infection [6]. In Tanzania, NTM species including *M. gordonae*, *M. smegmatis*, *M. fortuitum*, *M. phlei*, *M. flavescens* and *M. avium intracellulare* have been detected in milk samples collected from the coast region [7]. Studies conducted in Arusha, northern Tanzania found NTM to be more common than *M. tuberculosis* in patients with TB adenitis [8]. Moreover, invasive NTM infections in Tanzania due to *M. sherrisii* and *M. avium* complex sequevar *M. avium* complex-D has been diagnosed in HIV-infected patients [9], also NTM infection due to *M. intracellulare* has been found to be associated with non-HIV infected patients with a history of tuberculosis [10].

In the Serengeti ecosystem a very high interaction of humans-environment-livestock- wildlife exists, posing a risk for animal-and human-based contamination of the natural water sources and the transmission of NTM to other animals or humans [11]. However, little information is available on NTM species circulating in humans, domestic and wild animals and even fewer reports are available in Africa, although Botha *et al.*[12] have reported that the following NTMs have been found in human and animal specimens: *M. abscessus*, *M. asiaticum*, *M. avium*, *M. brasilienses*, *M. chelonae*, *M. elephantis, M. engbackii, M. farcinogenes, M. fortuitum, M. gilven, M. gordonae, M. heraklionense, M. hiberniae, M. intracellulare, M. interjectum, M. lentiflavum, M. marseillense, M. moriokaense, M. nonchromogenicum, M. palustre, M. palveris, M. paraffinicum, M. phlei, M. senegalense, M. simiae, M. sherrisii, M. sphagni, M. terrae* and *M. vulneris*.

The use of molecular techniques that target the 16S rRNA gene has been useful for diagnosis of NTM. This is particularly important in TB endemic regions where NTMs are underdiagnosed in TB patients due to limited availability of laboratory facilities [13]. Most health centres in developing countries depend on Ziehl-Neelsen (ZN) for diagnosis of tuberculosis which cannot distinguish *M. tuberculosis* complex (MTBC) from NTM [14],[15]. Diagnosis of pathogens using molecular diagnostic techniques is more rapid and improve accuracy in identification as compared to conventional methods which are time consuming [16]. Sequencing of the 16S rRNA gene in NTM can be applied in the speciation of bacteria, even those which are dead or are uncultivable [15],[16]. Therefore, we used this technique to determine species diversity of NTM in biological samples collected from humans, wildlife and livestock at the human-animal interface in the Serengeti ecosystem, Tanzania.

## Methods

### Study site

This study was conducted in the Serengeti ecosystem and surrounding districts in Bunda (S 2° 0' 0, E33° 49' 60), Serengeti (S2° 0' 0, E 34° 49' 60) and Ngorongoro (E35° and 36°E and S 2° and 4°) (Figure 1). The Serengeti ecosystem as previously described by Katale et al. [17] is comprised of Serengeti National Park (SNP), Ngorongoro Conservation Area (NCA), Loliondo Game Controlled Area (LGCA), Maswa Game Reserve and Ikorongo-Grumeti Game Reserve (IGGR). Livestock and wildlife in the ecosystem interact during grazing and drinking water increasing possibility of interspecies disease transmission or contamination of water sources, which could potentially lead to a zoonotic epidemic [17].

### Sampling of livestock and wildlife tissues

#### Livestock

Four hundred and nighty nine (499) tissues (mediastinal, retropharyngeal, pre-crural and pre-scapular lymphnodes, lungs and liver) with lesions suggestive of mycobacterial infection were collected from approximately 1200 slaughtered indigenous zebu cattle (*Bos indicus*) in the Serengeti (n = 420), Bunda (n = 600) and Ngorongoro (n = 180) abattoirs (Records from registry books, District Meat Inspection [2012-2013]). The slaughter houses were located in district headquarters where cattle were purchased from different local markets available in the districts and transported to district abattoirs for slaughter. Meat inspections were conducted with assistance of meat inspectors at the slaughter houses. To ensure that only cattle carcasses from the study site were sampled, the history of the origin of the cattle was taken from the owners. Tissues were selected based on presence of tuberculous lesions in organs and their associated lymph nodes. The anatomical sites of bTB suspected lesions for each carcass were recorded. With assistance from meat inspectors tuberculous organs and their associated lymph nodes were palpated, incised and preserved separately in sterile zip lock bags and packed in a cool box before processing for storage in liquid nitrogen containers.

#### Wildlife

One hundred and seven (107) wildlife tissues were sampled opportunistically from dead animals in the Serengeti ecosystem. Tissues sampled included road kill, where only intact organs were sampled (2 impala and 1 black-backed jackal), and animals that died from old age or disease. Forty three (43) tissues which included mediastinal, retropharyngeal, mesenteric lympnodes, lungs, kidney and liver were sampled during trophy and meat cropping in Maswa Game Reserve (MGR) as per the schedule of the Ministry of Natural Resources and Tourism, Tanzania. Tissue specimens were preserved in liquid nitrogen containers for further analysis. Ziehl-Neelsen (ZN) was performed on tissues that showed growth on LJ Media.

### Tissues processing and culture

Tissues of approximately 10 g were chopped into small pieces using sterile scalpel blades and forceps and transferred into stomacher bags containing 10 ml of sterile distilled water. Samples were then homogenized for 2 min using a blender (Stomacher 80 laboratory blender, Seward Medical, London, UK). The homogenates were transferred into universal containers, followed by addition of an equal amount of 3% oxalic acid to the homogenate in the universal container. The suspensions were left for 45 minutes at room temperature with occasional shaking before centrifugation at 3000 rpm (Mistral 1000 MSE, UK) for 15 min. The sediments were neutralized with 3-4 drops of 2% sodium hydroxide solution to which 3-4 drops of 0.1% phenol red indicator were added. The sediments were mixed before inoculation on Lowenstein Jensen (LJ) Media (BDH Chemicals Ltd, Poole, UK) containing pyruvate or glycerol and incubated at 37°C. LJ slopes were examined weekly for indications of macroscopic growth for 12 weeks. When growth was visible, smears were prepared, air dried, fixed with heat, stained with ZN and examined microscopically for the presence of acid fast bacilli (AFB).

### Sputum sample collection and preparation for culture

Sputum samples were collected from 472 hospitalized and out-patients who attended TB clinics in Bunda, Serengeti and Ngorongoro district hospitals. Sputum samples were collected in 50ml screw-cap Falcon tubes in the early morning. Both hospitalized and outpatients with symptoms suggestive of TB in the study area, new patients who had not yet been started on anti-tuberculosis treatment were considered for inclusion in the study. Digestion and decontamination of sputum samples were carried out by adding an equal volume of Cetyl Pyridinium Chloride (CPC) solution to the sputum samples. The specimens were left for 15 min at room temperature and concentrated by centrifuging at 4000rpm for 15 min. The supernatants were poured off into a splash proof container. Twenty (20) ml of distilled water was added to the sediments and the pellets were suspended by inverting the tubes several times before centrifugation at 3500 rpm for 15 min. The supernatant was poured off and the deposits were inoculated onto two slopes of LJ media containing glycerol or pyruvate by using sterile loops. Specimens were incubated and examined weekly for bacterial growth. Smears on microscope slides were prepared from the specimen sediments and thereafter stained by the Ziehl-Neelsen (ZN) stain.

### Extraction of DNA

Mycobacterial DNA was isolated from colonies of each isolate that were scraped from the surface of the LJ medium containing glycerol or pyruvate. The isolates were suspended into labeled cryovials containing 100μl of sterile distilled water. The suspensions were heated in a water bath at 80°C for 1 hour to inactivate the bacteria and then centrifuged at 3000 rpm (Mistral 1000 MSE, UK) for 5 min. The supernatants were stored at −80°C until further analysis.

### Mycobacterium genus typing

Multiplex PCR was performed using six primers as described by Wilton & Cousins et al. [18]. Briefly, six oligonucleotide primers namely MYCGEN-F 5’-AGA GTT TGA TCC TGG CTC AG-3’, MYCGEN-R 5’-TGC ACA CAG GCC ACA AGG GA-3’, which amplify a specific PCR product from the 16s rRNA gene of all know mycobacteria were used. MYCAV-R 5’-ACC AGA AGA CAT GCG TCT TG-3’ and MYCINT-F 5’-CCT TTA GGC GCA TGT CTT TA-3’ which amplify the hyper variable region of the 16S rRNA gene of *M. intracellulare* (MYCINT-F) and *M. avium* (MYCAV-R) respectively. Two primers (TB-F, TB-R) (TB-F 5’-GAA CAA TCC GGA GTT GAC AA-3’ and TB-R 5’-AGC ACG CTG TCA ATC ATG TA-3’ (TIB Molbiol® syntheselabor GmbH, Germany) which target for the MPB70 gene were used to specify *M. tuberculosis* complex from the mycobacteria. Briefly, one PCR reaction consisted of 2 μl DNA, 6.2 μl H_2_0 Qiagen®, 10 μl of master mix and 0.3 μl for each of the primers. Initially, DNA was denatured at 95°C for 10 min, annealing involved 35 cycles for 1 min, 0.5 min and 1 min at 95°C, 61°C and 72°C, respectively. Extension was done at 72°C for 10 min and cooling at 4°C infinitely at 4°C. In all cases of DNA amplification water was included as negative controls and *M. tuberculosis* complex DNA as positive controls in each run. Electrophoresis was run at 100 V in 1.5% agarose (3.75 g in 250 ml of 1 × TAE buffer) (Sigma®) gel and visualized under UV light. During electrophoresis, Ethidium bromide was used as a staining reagent. The PCR products were calibrated using hundred base pair (100 bp) DNA ladder, blue loading dye (Promega Corporation, Madison, USA).

### Amplification of DNA for Mycobacterial speciation of NTM

PCR amplification of mycobacterial DNA samples was done using 16S rRNA f- 5’ AGA GTT TGA TCC TGG CTC AG 3’ and 16S rRNA r- 5’ GCG ACA AAC CAC CTA CGA G 3’ primers. Each PCR reaction contained: 1 μl of DNA template, 2.5 μl of 10× buffer, 2 μl 25 mM MgCl_2_, 1 μl 10 mM dNTPs, 5 μl Q-buffer, 0.5 μl of each primer (50pmol/μl), 0.125 μl HotStarTaq DNA polymerase (Qiagen, Germany) in a 24.5 μl reaction with ddH2O. A negative control (no template) and a positive control (DNA template from *M. tuberculosis* H37Rv) were included to assay for contamination of the reagents and successful PCR amplification, respectively. Amplification was done by activating the Taq polymerase at 95’C for 15 min, followed by 45 cycles of 94°C for 30 s, 60°C for 1 min and 72°C for 30 s, followed by a final elongation step at 72°C for 10 min.

### Nucleotide sequencing of non-tuberculous mycobacteria

To identify mycobacterial species, polymerase chain reactions (PCRs) were conducted followed by sequencing of the 16S rRNA gene [19] using 1.1pmol/μl16S rRNA forward primer (16S-27f (5'- GWA TTA CCGCGG CKG CTG -3'). Sequencing was done using an ABI sequencer (Applied Biosystem Inc.) at the Central Analytical Facility (CAF) of Stellenbosch University, South Africa. Accession numbers and obtained sequences were edited and analysed using the Ribosomal Differentiation of Microorganisms (RIDOM) project (http://www.ridom-rdna.de) and the National Center for Biotechnology Information (NCBI) Blast sequence alignment tool (http://blast.ncbi.nlm.nih.gov).

### Ethical consideration

This study was approved by the Research Ethics committee of the Muhimbili University of Health and Allied Sciences (MUHAS)(Ref.MU/PGS/PhD/R/Vol.1), The National Institute for Medical Research (NIMR) (Ref. No. NIMR/HQ/R.8a/Vol. IX/1299), Tanzania, Tanzania Wildlife Research Institute (TAWIRI) and the Ministry of Natural Resources and Tourism, Tanzania (Ref. No. HA 403/563/01/74). Written informed consent was taken from human subjects and exclusion from the study is without any obligations on both sides.

## Results

The 606 tissue specimens collected from livestock and wildlife species showed that 5 specimens (0.8%) were identified as MTBC members and 43 specimens (7.1%) were characterized as NTM species, which consisted mostly of *M. intracellulare* (20 of the 43 samples, 46.5%). Other NTM species identified were *M. lentiflavum* isolated from 6 cattle specimens, 3 buffalo specimens, 1 Thompson gazelle specimen and 1 isolate from a warthog. *M. simiae*, *M. confluentis*, *M. neoaurum, M. nonchromogenicum*, *M. terrae* and *M. thermoresistibile* were isolated from cattle only (see Table 1). No growth was observed for the other 558 (92.1%) tissue specimens cultured on LJ medium. From the 472 human sputum samples, only 12 specimens (2.9%) were NTM positive which were *M. Chelonae*-*abscessus* group (n = 3*)*, *M. genavense*, *M. gilvum*, *M. gordonae*, *M. intermedium*, *M. poriferae* and *M. spaghni* (n = 2). No *M. avium* species were identified from human or animal specimens. Most of NTM species isolated in this study have been found in other countries such as Uganda, Kenya, Zambia and South Africa (Table 2). No report was found whether *M. genavense, M. poriferae* and *M. spaghi* have been isolated in these countries (Table 2). All members of the mycobacteria genus gave a PCR product of 1030 bp. *M. intracellulare* generated a PCR fragment of 850 bp in addition to the 1030 bp genus product. PCR fragments of 372 bp in addition to the 1030 bp product were generated by isolates from the *M. tuberculosis* complex.

**Table 1:** **Species diversity of NTM isolated from humans, livestock and wildlife based on sequencing of 16S rRNA gene**

NTM Species	Host
*M .lentiflavum*	6 cattle, 3 buffalo, 1 Thompson gazelle, 1 warthog
*M .simiae*	2 cattle
*M. chelonae-abscessus group*	3 human
*M. confluentis*	1 cattle
*M. fortuitum*	3 cattle, 1 human
*M. genavanse*	1 human
*M. gilvum*	1 human
*M. gordonae*	1 human
*M. intermedium*	1 human
*M. intracellulare*	17 cattle, 1 human, 1 Thompson gazelle, 1 baboon
*M. neoaurum*	2 cattle
*M. nonchromogenicum*	2 cattle
*M. poriferae*	1 human
*M. spaghni*	2 human
*M. terrae*	2 cattle
*M. thermoresistibile*	1 cattle

**Table 2:** **Comparison of species diversity of NTM in Tanzania with other Sub-Saharan African countries**

NTM spp	Culture source	Host/source	Country	Reference
*M .lentiflavum*	Sputum, animals tissues	Human, animals	Zambia, Uganda, Kenya, Zambia	Muyoyeta et al. [20], Asiimwe et al. [14], Mijele et al. [21], Buijtels et al. [22], Buijtels et al. [23], Panagiotou et al. [24]
*M .simiae*	Water, soil	Animals, environment	Uganda, South Africa	Kankya et al. [25], Gcebe et al. [26]
*M. chelonae*	Sputum, animal tissues	Human, animals	Zambia, Uganda	Panagiotou et al. [24], Muwonge et al. [27]
*M. confluentis*	Soil, animal tissues, sputum	Animals, environment, human	South Africa, Ethiopia	Gcebe et al. [26], Workalemahu et al. [28]
*M. fortuitum*	Sputum, soil, water, faecal, animal tissues	Human, environment, animals	Zambia, Uganda, Kenya, Ethiopia	Muyoyeta et al. [20], Kankya et al. [25], Nyamogoba et al. [29], Berge et al. [30], Workalemahu et l. [28], Buijtels et al. [23], Panagiotou et al. [24], Muwonge et al. [27]
*M. genavanse*	*	*	*	*
*M. gilvum*	Sputum	Human	Zambia	Buijtels et al. [23]
*M. gordonae*	Sputum, water, soil, animal tissues	Animals, Human, environment	Zambia, Uganda, South Africa, Kenya, Ethiopia	Muyoyeta et al. [20], Kankya et al. [25], Asiimwe et al. [14], Gcebe et al. [26], Mijele et al. [21], Berge et al. [30], Amen et al. [31], Buijtels et al. [23], Panagiotou et al. [24], Muwonge et al. [27]
*M. intermedium*	Soil, water	Animals, environment	South Africa	Gcebe et al. [26]
*M. intracellulare*	Water, soil, sputum, animal tissues	Human, environment, animals	Uganda, Kenya, Ethiopia, Zambia	Kankya et al. [25], Asiimwe et al. [14], Nyamogoba et al. [29], Mijele et al. [21], Berge et al. [30], Buijtels et al. [32], Buijtels et al. [23], Panagiotou et al. [24]
*M. neoaurum*	Animal tissues	Animals	Uganda	Muwonge et al. [27]
*M. nonchromogenicum*	Water, soil, animal tissues, swabs	Animals, environment	Uganda, South Africa, Ethiopia	Kankya et al. [25], Gcebe et al. [26], Berge et al. [30]
*M. poriferae*	*	*	*	*
*M. spaghni*	*	*	*	*
*M. terrae*	Water, animal tissues, soil, sputum	Animals, environment, human	South Africa, Ethiopia, Zambia, Uganda	Kankya et al. [25], Gcebe et al. [26], Workalemahu et al. [28], Buijtels et al. [23], Muwonge et al. [27]
*M. thermoresistibile*	Soil	Environment	South Africa	Gcebe et al. [26]

*No report is available from other countries.

## Discussion

We found the overall prevalence of NTM in livestock and wildlife to be 7.1%, which is not far from results estimated based on comparative skin tests conducted in the same ecosystem in indigenous cattle. This study showed non-specific reaction (atypical mycobacterium) to avian and bovine tuberculin purified proteins derivatives (PPDs) to be 10.6% in indigenous zebu [17]. However, we were unable to find either NTM or MTBC in a high proportion (92.1%) of tissues with apparent tuberculous lesions, indicating that the lesions might be caused by other pathogens or there was a failure of egg based LJ medium to culture mycobacteria from these lesions. Liquid media is more efficient for recovery of mycobacteria than egg based solid medium [20]. A report by McCarthy et al. [21] found large differences in recovery of NTM between solid or liquid media, a study that cultured NTM from patients with HIV. However, in the present study liquid medium was not used for isolation of mycobacterium. Neither, was the HIV status of human TB suspected patients available to correlate the NTM infection with HIV status.

This study has shown that, 16 species of NTM were isolated from humans, indigenous cattle and wildlife, in the Serengeti ecosystem, of which the majority were *M. intracellulare*, *M. lentiflavum and M. fortuitum*. Other NTM species including *M. cholenae*-*abscessus* group*, M. simiae, M. neoaurum, M. nonchromogenicum, M. spaghni, M. terrae, M. confluentis, M. genavense, M. gilvum, M. gordonae, M. intermedium, M. poriferae* and *M. thermoresistibile* were isolated at a lower rate, ranging between 5.5% and 1.8%. Comparison of the NTM species isolated in this study indicate that most of them have previously been found in humans, animals, or the environment in Ethiopia, Kenya, Uganda, South Africa and Zambia (Table 2). In the present study, given the nature of sample acquisition for both cattle and wildlife specimens, isolation of *M. gordonae* and *M. terrae* complex as environmental contaminants [22] might occur. Thus there is unjustifiable potential for specimens’ environmental contamination which might obscure our findings. *M. genavense, M. poriferae* and *M. spaghni* are not common or have not previously been isolated in East Africa (Table 2). In contrast to our study where all NTM species were isolated from sputum samples and animals tissues. Previous studies found *M .simiae*, *M. fortuitum*, *M. gordonae*, *M. intermedium*, *M. intracellulare*, *M. nonchromogenicum* and *M. terrae* in soil and water (Table 2). The correlation of NTM between soil on one hand and animals on the other hand indicates that NTM are readily exchanged between animals and environments [23]. However, in our study no any environmental sample was taken to correlate between NTM species found in animals and environments.

*M. intracellulare*, which is a member of *Mycobacterium avium* complex (MAC) was the most frequently isolated species, consistent with a report from study of NTM in Uganda, South Africa and Australia [24],[25]. However, this finding differs from other studies in Kenya, South Africa and Uganda where *M. mucogenicum*, *M. terrae* and *M. fortuitum* were the most frequently isolated species respectively [14],[16]. The high prevalence of *M. intracellulare* is of concern in this setting, given a high HIV prevalence and the ability of *M. intracellulare* to cause pulmonary and extrapulmonary TB in such individuals [26],[27] such as has been reported in Kenya [28]. In the current study, *M. intracellulare* species were isolated from human, cattle and wildlife.

We also found *M. fortuitum*, *M. chelonae*-*abscessus* group, *M. genavense*, *M. gilvum*, *M. gordonae*, *M. intermedium*, *M. poriferae* and *M. spaghni* in TB suspect patients who had clinical signs suggestive of tuberculosis. However the role of these NTM in TB disease causation as well as possible influence in the diagnostic tests for tuberculosis is not known in our cases. This finding is consistent with previous studies where these species were isolated from respiratory samples from patients with chronic bronchitis, pulmonary TB, sub-acute pneumonia and healed pulmonary TB [29]-[32]. In Uganda, *M. fortuitum* was the most prevalent NTM species isolated from respiratory secretions in infants and adolescence [13].

We isolated from tuberculous lesions of indigenous cattle a number of NTM species which include *M. simiae*, *M. confluentis*, *M. neoaurum, M. nonchromogenicum*, *M. terrae* and *M. thermoresistibile.* Apparently these animals had neither *M. bovis* nor other MTBC and it may be important to examine the role of these NTM in disease causation. It is also potentially important to test the effect of exposure of animals to NTM on the specificity and/or sensitivity of bovine tuberculosis (bTB) diagnostic test [33] such as skin test and interferon gamma assay due to potential cross-reactivity.

Lastly, we detected a low prevalence of *M. nonchromogenicum* in cattle and *M. lentiflavum* from indigenous cattle and wildlife. *M. nonchromogenicum,* part of the *M. terrae* comple*x,* has also been isolated from nasal mucus from cattle in a herd infected with bTB [34] and in immunosuppressed individuals with pulmonary disease [35]. Infections due to *M. terrae* complex are rare [36]. However, *M. nonchromogenicum* has been associated with bacteremia in AIDS patients [25], lung infection in humans and has been detected from patients with chronic skin ulcers unresponsive to antibiotic treatment, arthritis [37], tenosynovitis [38], pulmonary infection [37],[39] and meningitis [40], signifying its public health and veterinary importance. Together with *M, terrae*, *M. nonchromogenicum* is widely distributed in Africa although proportions differ in different countries [23].

The ubiquitous nature of NTM suggests value for investigation of their clinical relevance as well as effects on interpreting other positive TB test results, for example, we do not know what the influence of NTM on immunity and immunological based TB diagnostic tests, given the fact that mycobacteria share many common antigens.

## Conclusion

In conclusion, there is a very wide range of NTM species from humans and animals in the Serengeti ecosystem and understanding their transmission dynamics will require a one health approach involving sampling and sequencing of samples from livestock, wildlife, humans and their environment. Furthermore, there is a need to investigate the influence of NTM in disease causation and efficacy on screening and diagnostic tests.

## Authors’ original submitted files for images

Below are the links to the authors’ original submitted files for images. Authors’ original file for figure 1
