# Supplementary figures and images for: Species diversity of non-tuberculous mycobacteria isolated from humans, livestock and wildlife in the Serengeti ecosystem, Tanzania
Source: BMC Infect Dis. 2014 Nov 18;14:616. doi: 10.1186/s12879-014-0616-y (PMC4239340; doi:10.1186/s12879-014-0616-y)

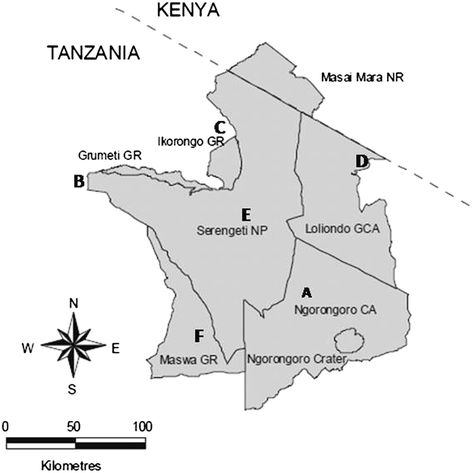

Supplement: Supplementary file 1 — Authors’ original file for figure 1 [file 12879_2014_616_MOESM1_ESM.gif]
